# Penetrating Cardiac Injury: A Review

**DOI:** 10.5812/traumamon.3461

**Published:** 2012-05-26

**Authors:** Mohd Lateef Wani, Ab Gani Ahangar, Shadab Nabi Wani, Ifat Irshad, Nayeem Ul-Hassan

**Affiliations:** 1 Department of cardiovascular and thoracic surgery SKIMS Soura, Soura, India

**Keywords:** Heart Injuries, Commotio Cordis

## Abstract

Cardiac injury presents a great challenge to the emergency resident because these injuries require urgent intervention to prevent death. Sometimes serious cardiac injury may manifest only subtle or occult symptoms or signs. As there is an epidemic of cardiac injuries in Kashmir valley due to problems of law and order, we herein present a review on management of such injuries.

## 1. Introduction

Cardiovascular penetrating injuries are common in military conflicts in large numbers. As a result most of the data are from major wars of the past century ( World War 1, World War II, Korean Conflict, Vietnam War, Gulf war I and II, as well as civilian wars in the Middle East, Yugoslavia, Russian Republic, Kashmir and Central Africa. Military injuries are seen almost exclusively in young males usually free from chronic vascular diseases. Injuries are often due to high velocity projectiles which cause extensive damage and disruption of collateral circulation.

In the early 18th century Boerhaeve labeled all penetrating cardiac trauma as fatal (1). Billroth stated “The surgeon who should attempt to suture a wound of the heart should lose the respect of his colleagues” (2). Rehn succeeded in repairing a 1.5 cm right ventricular stab wound procedure named cardiorraphy (3, 4). Hill did the first successful cardiorraphy in the USA (5).

The first successful pericardiocentesis for a cardiac wound was accomplished by Larry, the surgeon to Napoleon (3, 4).

In 20^th^ century emergency operative intervention was used as definitive treatment for cardiac trauma instead of pericardiocentesis (2-4).

**Projectile injuries to the heart can conveniently be divided into**

1. Contusion of myocardium
2. Laceration and puncture of chambers
3. Disruption/rupture of valves and leaflets
4. Disruption/perforation of septum.
5. Injury to coronary vessels

True incidence of cardiac trauma in the military is difficult to ascertain. On the battle field many cardiac injuries are fatal. Dixon and McEwen in 1916 proclaimed, “Probably nearly all cardiac wounds produce death from haemorrhage too quickly to allow the patient being removed alive even to a short distance from the battle field.”(6)

Harkin in World War II reported a unique experience of removing foreign bodies from the heart and adjacent vessels in 134 patients (7) .Valle (1955) reported an incidence of 4.2% of injuries to heart and mediastinum (8). Gielchinsky and McNamara (1970) reported an incidence of 2.8% in the Vietnam war (9).

The etiology of heart wounds is of extreme importance. Small pericardial and myocardial wounds with tamponade may be successfully treated by pericardiocentesis. On the other hand, larger wounds of pericardium and myocardium caused by bullets should be managed by immediate thoracotomy and cardiorrhaphy. Projectile emboli to the heart represents an unusual type of injury. There are numerous case reports to attest to the professional interests and challenge in managing these types of injuries. Morton and colleagues (1971) reported three cases in which a bullet embolized from a vein of the lower extremity to the right ventricle (10).

The majority of heart wounds treated by military surgeons have resulted from fragment wounds. Gunshot wounds of the heart are usually fatal. Low velocity projectiles such as small fragments from anti-personnel and anti-tank mines, mortars, grenades, rockets and bombs are responsible for the majority of wounds which result in pericardial tamponade. However, more extensive damage to the heart and the great vessels, due to high velocity wounds usually result in immediate exsanguinations. Both early diagnosis and prompt management are of paramount importance for salvaging such patients.

It is estimated that 80-90% of patients with gunshot wounds of the heart do not reach the hospital (11).The standard approach has been to repair the lacerations using mattress sutures controlling hemorrhage with a finger on the heart. Emergency temporary control of hemorrhage from cardiac laceration can be achieved with the use of a skin stapler (12). Following stabilisation of the patient, the staples can be removed after definitive suture repair is performed in the operating room. Hemostatic sealants such as Floseal, etc., offer significant promise as an additional tool in the surgical armamentarium when dealing with laceration to the heart (13). Regardless of the approach used, care must be taken to avoid injury to coronary arteries.

The most common chamber involved in projectiles injuries of the heart is the right ventricle obviously due to its anatomical location. Most of the series conclude that the right ventricle is the most common chamber involved (14, 15).However, World War II combat experience varies somewhat in that the left ventricle was involved more often than the right ventricle (16). This is an exception, however, because the location of cardiac wounds in the Vietnam experience again emphasized the predominance of right ventricle injury.

There are three primary physiological disturbances associated with cardiac trauma:

1. Hemorrhage,
2. Pericardial tamponade,
3. Cardiac failure,

Every cardiac injury has some degree of hemorrhage, which may vary from hemorrhage into the myocardium associated with contusion to exsanguinating hemorrhage into the thoracic cavity or outside the cavity, associated with penetrating or perforating wounds. The later type is usually associated with early mortality.

Impaired cardiac function from penetrating trauma is unusual and is seen only in rare instances, such as trauma to a heart valve, the conduction bundle or a major coronary artery.

Frequently, the dominant physiological injury in surviving patients is cardiac tamponade. Cardiac tamponade provides an early opportunity for survival; however, it also contributes very rapidly to mortality associated with cardiac wounds. The tamponade can delay or stop bleeding from cardiac laceration, allowing the patient to survive long enough for definitive therapy. Cardiac tamponade occurs quickly because the normal pericardium can accommodate only 100 to 250 ml of blood. There is a progressive fall in cardiac output as the intrapericardial pressure rises with expanding cardiac tamponade. The ultimate restriction of cardiac output results from the external compression to prevent diastolic filling of the ventricles. The release of cardiac tamponade will increase cardiac output, help to restore normal circulation and alleviate anoxia.

A high index of suspicion for cardiac trauma is extremely important. Because pericardial tamponade is encountered frequently by an average physician, the diagnosis can often be overlooked, with a resultant tragic outcome because tamponade is a true medical emergency. Beck’s triad is associated with cardiac tamponade,decreased arterial pressure, elevated venous pressure manifested by distended neck veins , and muffled heart sounds ;however, not all of these signs may be apparent . The patient is frequently weak, restless and thirsty with other signs frequently associated with a patient in shock. Chest pain is usually not severe. Immediately upon admission to an emergency area, it is of paramount importance that all clothing be removed. All patients with wounds of the pericardium, chest, back or upper abdomen should be examined for signs of cardiac tamponade. It must be remembered that with reduced blood volume, hypotension with pulsus paradoxus and jugular venous distension may appear only after volume deficit has been corrected.

## 2. Diagnosis of Projectile Cardiac Injuries

The diagnostic approach to patients with cardiovascular injuries from projectiles has evolved over 60 years, largely based on military experience. Physical diagnosis of these cardiac injuries is usually obvious. On the other hand, cardiac tamponade may be confused with pulmonary embolism, cardiac failure, and over-transfusion. The following sections can aid in this diagnostic dilemma.

## 3. Central Venous Pressure

Accurate measurement of central venous pressure is an important early diagnostic maneuver. If the venous pressure is above 15 cm of saline solution the test is essentially diagnostic. However falsely elevated central venous pressure may be due to straining, shivering or malpositioned catheter.

## 4. Chest Radiography

Chest radiograph will usually not show cardiac enlargement with tamponade because the pericardium does not easily distend. On the other hand, even under battlefield conditions there may be an increased cardiac diameter that can be appreciated. Hemothorax is a common finding, particularly with concomitant injuries. Anteroposterior and lateral roentgenograms of the chest should be obtained to identify the location of foreign bodies. If the missile or fragment entered the heart and the point of exit cannot be found, peripheral embolization of the projectile should be highly suspected .This may be particularly true if the follow-up radiograph does not show the foreign body that was previously apparent in the area of the heart. If there is an associated pericardial tear, hemothorax may be obvious, obscuring cardiac borders. A pneumopericardium may only become obvious after tube thoracostomy.

## 5. Computed Tomography

Computed tomography of the chest can show the hemopericardium giving detailed information of associated pulmonary injury and hemothorax. It can also show the accurate location of the foreign body if present.

## 6. Electrocardiogram

The electrocardiogram has been of some assistance. Yao and co-workers 1968 emphasized that muffled heart sounds and elevation of the S-T segment in precordial leads may support the diagnosis of pericardial penetration. As should be anticipated specific wounds to the coronary arteries and conduction area of the heart may be associated with specific changes on the electrocardiogram.

## 7. Echocardiography

This noninvasive test can aid in diagnosis of pericardial tamponade besides giving an idea of function of different cardiac chambers.

## 8. Treatment:

The etiology of heart wounds is of extreme importance. Small pericardial and myocardial wounds with tamponade may be successfully treated by pericardiocentesis. On the other hand, larger wounds of the pericardium and myocardium caused by bullets should be managed by immediate thoracotomy and cardiorrhaphy. It must be remembered that patients with projectile cardiac injury do not give us enough time to call the attending surgeon for these investigations. Usually the patient is directly shifted to the operating environment and explored. Prompt decision of operative intervention can make the difference between life and death. A high index of suspicion for cardiac injury, understanding the mode of presentation and rapid diagnosis affects the outcome. Proper resuscitation and control of bleeding is a must in these cardiovascular injuries. Hence it is concluded that there should be an aggressive approach when dealing with projectile cardiovascular injuries. Early intervention and prompt resuscitation should be done. No time should be wasted for time consuming investigations.
